# Rapid Epithelial Ingrowth Following Laser In Situ Keratomileusis due to Screwdriver Trauma

**DOI:** 10.1155/crop/5271498

**Published:** 2026-05-20

**Authors:** Yuhan Kuo, Takashi Ono, Yukako Taketani, Mikiko Kimakura, Tetsuya Toyono, Makoto Aihara, Takashi Miyai

**Affiliations:** ^1^ Department of Ophthalmology, University of Tokyo Hospital, Tokyo, Japan, u-tokyo.ac.jp

**Keywords:** corneal epithelium, corneal injury, LASIK, ocular infection, ocular injury

## Abstract

**Purpose:**

The purpose of this study is to report a unique case of rapid epithelial ingrowth following laser in situ keratomileusis (LASIK) flap trauma due to a flat blade screwdriver.

**Observations:**

A 36‐year‐old woman who had undergone LASIK 9 years ago experienced ocular trauma with a flat‐blade screwdriver during construction work. She was referred to our hospital with complaints of blurred vision and pain in the right eye and was prescribed antibiotic eye drops. Treatment improved the corneal epithelial defect within 3 weeks of the injury; however, epithelial ingrowth rapidly progressed beneath the LASIK flap 3 months after the trauma. The epithelial ingrowth reached the pupil area 10 months postinjury; nevertheless, the patient′s corrected visual acuity remained stable (1.2), and she continues to be followed up.

**Conclusion:**

Post‐LASIK trauma may lead to epithelial cell ingrowth at the interface of the corneal flap. Thus, careful long‐term follow‐up is essential, particularly following post‐LASIK corneal injury.

## 1. Introduction

Laser in situ keratomileusis (LASIK) is a refractive surgery used for myopia [1]. Although LASIK has been shown to be an effective ocular surgery for long‐term myopia correction, various complications, including regression and ocular infections, have been reported [2]. Epithelial ingrowth is a rare complication in which the corneal epithelial cells invade and proliferate beneath the LASIK flap [3]. Generally, epithelial cells slowly invade and develop from the wound to the space between the corneal stroma and the LASIK flap. However, because the flap is not directly sutured, it can be easily detached by ocular trauma. Therefore, if a patient sustains an ocular injury and the corneal flap is dislocated, corneal epithelial cells easily proliferate at the interface. Reattachment may be challenging if the detached flap surface is not smooth.

Screwdrivers can cause penetrating or perforating ocular injuries that are classified as open‐globe injuries. Penetrating injuries are formed when the screwdriver penetrates the eye but does not create an exit wound, and perforating injuries are formed when the screwdriver creates entrance and exit wounds. Even if the driver does not puncture the eye, sharp injuries can occur to the cornea, conjunctiva, and sclera on the eye surface. Furthermore, flathead screwdrivers are often contaminated with machine oil or soil, resulting in infectious keratitis. To date, there have been no clinical reports of ocular trauma in post‐LASIK eyes caused by a flat‐blade screwdriver.

Here, we report a rare case of rapid epithelial growth following ocular trauma due to a flat‐blade screwdriver.

### 1.1. Case Presentation

A 36‐year‐old woman was referred to the Department of Ophthalmology of the University of Tokyo Hospital with a chief complaint of blurred vision and pain in the right eye. Three weeks before the visit, the patient had been stabbed in the right eye using the tip of a flat‐blade screwdriver during a construction job. The patient visited the ophthalmology department of a neighboring physician and was treated with eye drops containing 1.5% levofloxacin and 0.5% cefmenoxime, and ointments containing 1% pimaricin and 0.3% ofloxacin as treatment by the first doctor. Once the infection was under control, the patient was referred to our hospital because of gradual inflammation recurrence. The patient had undergone bilateral LASIK 9 years prior and had no preoperative ocular conditions other than myopia. The patient had no medical history, internal medication, or allergies. At the first visit, the patient′s right eye demonstrated severe ciliary hyperemia, corneal edema, and inflammation in the anterior chamber (Figure 1a). Three corneal epithelial defects were noted with cellular infiltration extending to the area under the flap (Figure 1b,c). At the time of presentation, the LASIK flap was not completely detached and was attached to its original position on anterior segment‐optical coherence tomography (AS‐OCT) (Figure 1c). Visual acuity was 0.8p (0.9 × S − 0.50 D) in the right eye, 1.2 in the left eye, and intraocular pressure was 9.0 mmHg in the right eye and 15.0 mmHg in the left eye. Slit‐lamp examination showed no abnormal results in the anterior segment of the left eye. After scraping the lesion for culture assessment of the infected corneal lesion, topical treatment with 1.5% levofloxacin eight times daily, 0.5% cefmenoxime eight times daily, 0.3% tobramycin eight times daily, 5% pimaricin eye drops four times daily, and 0.3% ofloxacin eye ointment twice daily was initiated.

Figure 1Images of the anterior segment following ocular screwdriver trauma. (a) A photo of the anterior segment of the eye after ocular screwdriver trauma. Cell infiltration of the cornea with hyperemia is noted. The corneal flap was not dislocated. (b) A photo of the anterior segment with fluorescein staining of the cornea. Three areas with epithelial defects were clarified with staining (white arrows). (c) Images of the anterior segment‐optical coherence tomography. Corneal edema and cell infiltration are observed on the central ocular surface (white arrowhead). The focus was on reaching the area under the corneal flap.

**Figure:**
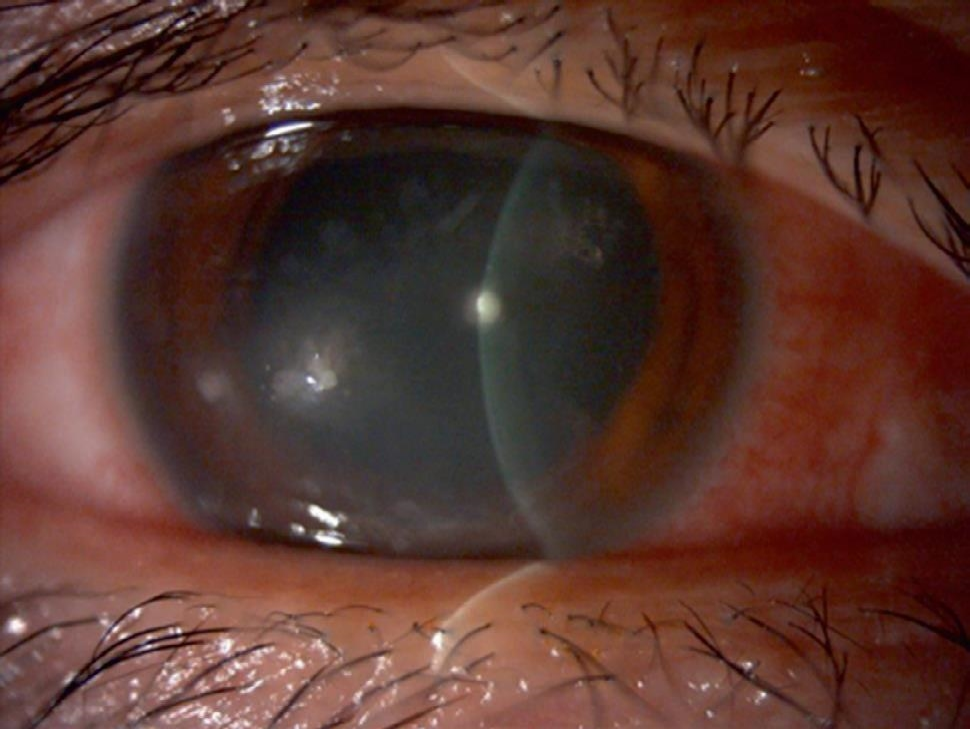
(a)

**Figure:**
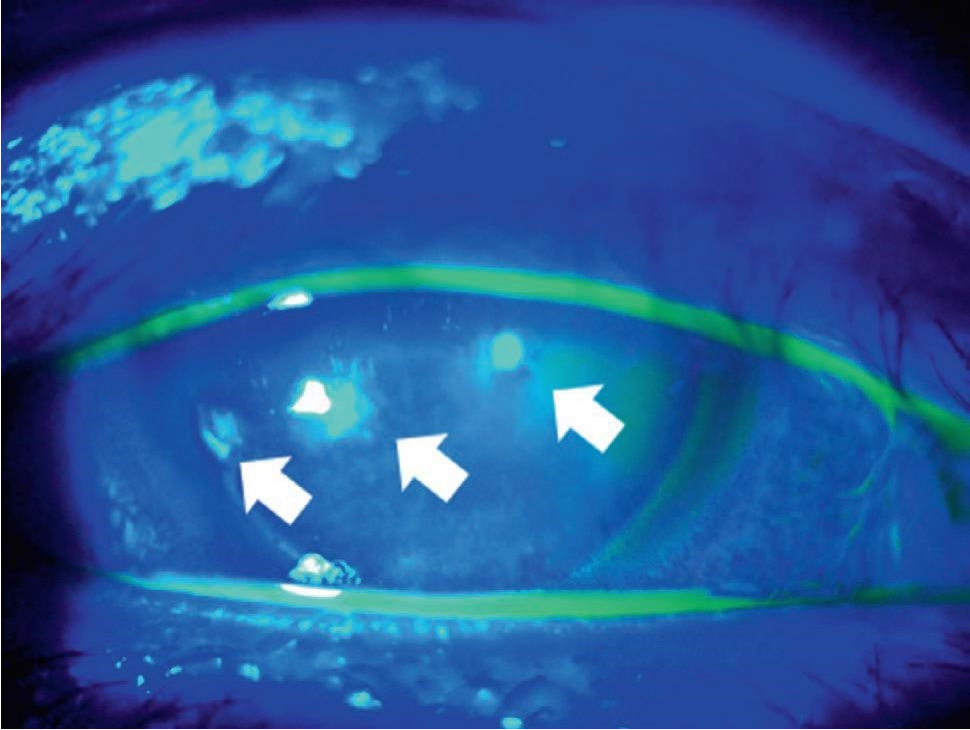
(b)

**Figure:**
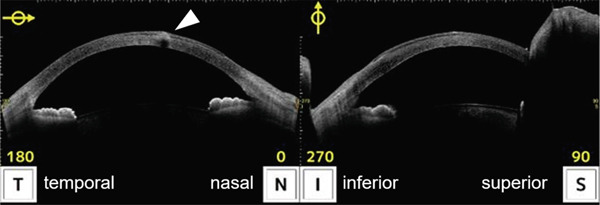
(c)

One week after treatment, cellular infiltration at the infected foci had decreased and the corneal epithelial defect had shrunk. Visual acuity improved to (1.0p × S + 0.25D: C − 1.50 D A × 60^°^). The culture test findings were negative. Given that no fungi were detected, pimaricin eye drops were discontinued, and cefmenoxime eye drops were discontinued a week later. Three weeks following the first visit, the epithelial defect disappeared, leaving only cellular infiltration (Figure 2a,b). We discontinued cefmenoxime and tobramycin eye drops, decreased levofloxacin eye drops to four times a day, and started 0.1% fluorometholone eye drops four times a day.

Figure 2Images of the anterior segment following topical treatment for keratitis. (a) Image of the anterior segment 3 weeks after the initial visit. Corneal scarring and cell infiltration decreased but slightly remained in the three areas. (b) Photograph of the anterior segment showing fluorescein staining of the cornea. The corneal epithelial defects disappeared. (c) Anterior segment image obtained using scattering illumination. Epithelial ingrowth between the corneal flap and stroma is noted as white scarring (white arrows).

**Figure:**
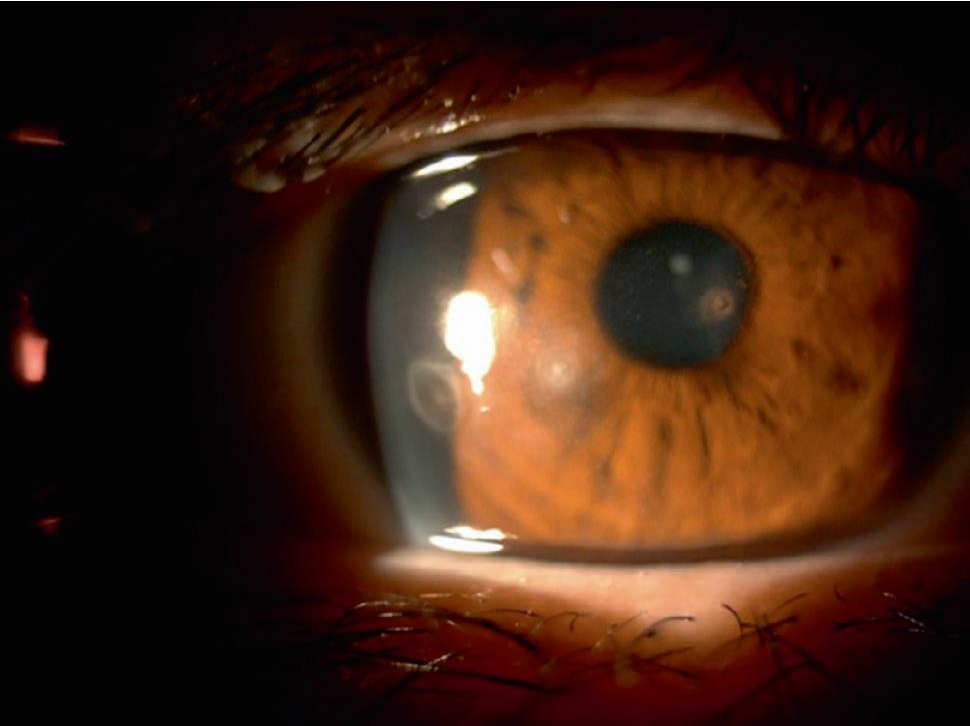
(a)

**Figure:**
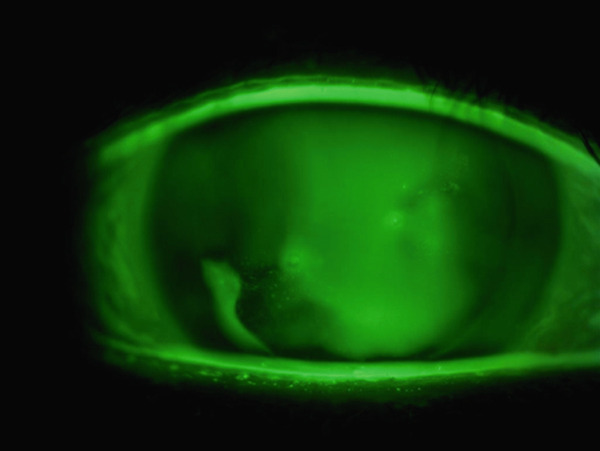
(b)

**Figure:**
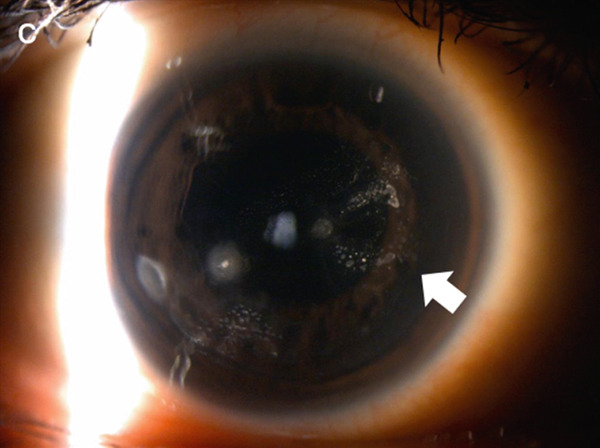
(c)

Infectious keratitis did not recur after initiating fluorometholone, and the cellular infiltration of the infected foci gradually disappeared and scarred. However, epithelial ingrowth began to appear under the nasal LASIK flap at the 3‐month visit (Figure 2c). Topical fluorometholone four times a day was continued, and epithelial ingrowth progressed inside the flap interface from the nasal to the temporal side and invaded the pupil area. Epithelial growth extension was confirmed using AS‐OCT at 3, 6, and 10 months following the injury (Figures 3a, 3b, and 3c). Fortunately, it did not affect the visual axis, and BCVA was maintained at Vd = (1.2 × S + 0.75 D : C − 1.00 D A × 135) 10 months after the injury. Therefore, we did not perform surgical intervention. The intraocular pressure was 17.5 mmHg at the last observation, and no complications of long‐term steroid usage were noted. No recurrence of infectious keratitis was observed.

Figure 3Images of the anterior segment optical coherence tomography of eyes with epithelial ingrowth following ocular trauma. (a) Images of the eyes 3 months after injury. Epithelial ingrowth is observed under the flap from the nasal side (red circle). (b) Images of the eyes 6 months following injury. An increase in the epithelial ingrowth under the flap on the temporal side is illustrated (red circle). (c) Images of the eyes 10 months after injury. The increase in epithelial ingrowth slightly continued and invaded the central cornea (red circle).

**Figure:**
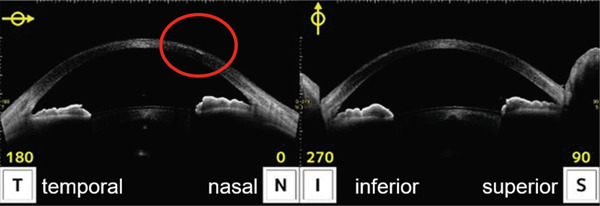
(a)

**Figure:**
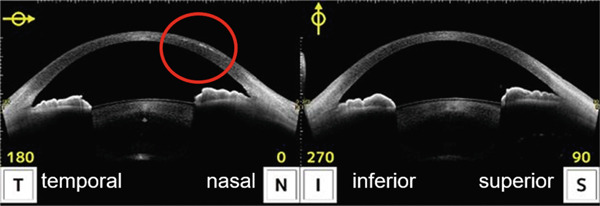
(b)

**Figure:**
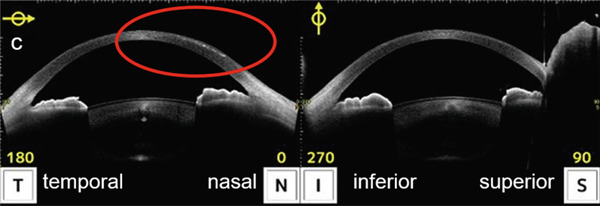
(c)

## 2. Discussion

Ocular trauma following LASIK can occur at any time after surgery because the corneal flap is not directly sutured and is not firmly attached to the corneal stromal bed. Different trauma types can affect the eyes following LASIK, including blunt trauma, sharp instrument trauma, and airbag deployment in car accidents. Flap dislocation, edema, and tears could occur due to instability of the corneal flap and the interface between the flap and corneal stroma. Surgical flap repositioning is necessary for patients with flap dislocation, and the procedure includes lifting, irrigating, repositioning, and suturing the dislocated flap [4]. Simultaneously, topical antibiotic administration is required to prevent and treat infectious keratitis. The interface between the corneal flap and the stromal bed is inside the stroma where topical medication is hard to reach; therefore, once infectious keratitis occurs, treatment with topical antibiotics that do not directly reach the infectious focus is challenging. In the present case, a flat‐blade screwdriver caused ocular trauma, and three epithelial defect areas were noted. The infection was stabilized with topical antibiotics, including levofloxacin, cefmenoxime, tobramycin, and ofloxacin, and infection of the corneal interface, did not occur. The prognosis of post‐LASIK ocular trauma is typically good, with prompt and appropriate treatment options. Following flap repositioning, 78% of the eyes achieved an uncorrected distance visual acuity of 20/20 or better [5]. Our patient also had good visual acuity after the injury.

Epithelial ingrowth following LASIK is an uncommon complication and is reported in 1.2%–3.9% of cases [6, 7], Among them, 0.92%–3.2% are clinically significant cases necessitating treatment, not only when the visual axis is involved but also when peripheral ingrowth causes flap elevation or irregular astigmatism [8] The condition of the overlying flap should be monitored closely, as dense epithelial growth can compromise nutrient supply and lead to flap thinning or melting [8]. Posttrauma cases have not been particularly quantified in the literature; however, ocular trauma could be a cause of epithelial ingrowth following LASIK [9]. In the present case, the patient experienced ocular trauma due to a flat‐blade screwdriver that caused deep keratitis to extend to the LASIK flap, and epithelial ingrowth progressed after injury. The representative clinical presentations of epithelial ingrowth include blurred vision, foreign body sensation, and visual disturbances. The reasons why epithelial ingrowth may cause visual disturbances include direct cell intrusion into the visual axis and regular and irregular astigmatism induction. Peripheral epithelial ingrowth may occur but does not always affect visual outcomes if it does not obscure the visual axis [10].

Epithelial ingrowth is classified into three grades: Grade 1, limited invasion within 2.0 mm of the flap edge with no associated visual changes; Grade 2, invasion of at least 2.0 mm from the flap edge but normal edge anatomy; and Grade 3, marked progression > 2.0 mm from the flap edge, often with anatomical abnormalities of the edge [11]. Regarding management, no treatment is required and careful observation should be performed for mild nonprogressive cases (Grade 1). Frequent monitoring is recommended to ensure that ingrowth does not progress. In contrast, flap lift and mechanical debridement are performed depending on the clinical symptoms in cases of progressive ingrowth (Grades 2 and 3). It involves lifting the LASIK flap and scraping off epithelial cells from the stromal bed and flap underside. For adjunctive treatments, ethanol application and mitomycin C are used to prevent cell proliferation [12, 13]. Suturing the LASIK flap after debridement can help prevent recurrence, particularly in severe or recurrent cases [14]. Additionally, fibrin glue is used on the edge combined with mechanical debridement and flap suturing to reduce recurrence [15]. Our case was categorized as Grade 2, and we selected it for observation because the patient maintained good visual acuity without any discomfort.

In conclusion, we encountered a case of epithelial ingrowth following LASIK that rapidly progressed after an ocular injury caused by a flat‐blade screwdriver. Appropriate eye protection when using tools and proper handling and storage of screwdrivers are essential to prevent these types of injuries, especially for eyes after LASIK.

## Author Contributions

All authors attest that they meet the current ICMJE criteria for authorship.

## Funding

No funding was received for this manuscript.

## Ethics Statement

This study was conducted in accordance with the principles of the Declaration of Helsinki and was conducted in the Department of Ophthalmology of the University of Tokyo Hospital. The Institutional Review Board of the University of Tokyo Hospital approved this study (Approval No. 2217).

## Consent

The patient provided informed consent for publication of the details of their medical case.

## Conflicts of Interest

The authors declare no conflicts of interest.

## Data Availability

Data are available on request due to privacy/ethical restrictions.
